# Not All in the Same Boat. Socioeconomic Differences in Marital Stress and Satisfaction During the Covid-19 Pandemic

**DOI:** 10.3389/fpsyg.2021.635148

**Published:** 2021-03-31

**Authors:** Maria Nicoleta Turliuc, Octav Sorin Candel

**Affiliations:** Department of Psychology, Faculty of Psychology and Educational Sciences, Alexandru Ioan Cuza University of Iaṣi, Iaṣi, Romania

**Keywords:** stress, marital satisfaction, socioeconomic status, gender differences, longitudinal study, COVID-19

## Abstract

The Covid-19 pandemic is a global threat that affects a large part of the population, but the risks associated with it are higher for some people compared with others. Previous studies show that lower socioeconomic status (SES) is associated with more chronic stress and less marital satisfaction. Thus, the uncertainty caused by the pandemic might greatly affect those who were already vulnerable. This longitudinal study explores the extent to which stress originated outside (external) and inside (internal) the relationship is associated with marital satisfaction during the Covid-19 pandemic and whether the associations are different based on the socioeconomic status of the participants. The study was conducted at two points in time (first, immediately after the national lockdown was instituted; second, after the lockdown ended) with a sample of 144 married Romanian couples. We used the Actor-Partner Interdependence Model with Mediation and multi-group SEM analysis. Higher levels of external stress were associated with subsequent lower marital satisfaction for women with higher SES. For the couples with lower SES, men's level of internal stress during the first assessment mediated the relationship between their higher level of external stress at the first time point and their partner's lower marital satisfaction during the second assessment. Our results show that men and women respond differently during a crisis and that couples with lower SES are more prone to greater stress and lower levels of marital satisfaction. We finally suggest that the therapists, health professionals, policy makers, and researchers should take into account the existing vulnerabilities of a couple when offering psychological and health services during the Covid-19 pandemic.

## Introduction

In Romania, the first case of the novel coronavirus (Covid-19) was confirmed on the 26th of February 2020 (Ceauşu, 2020). Since then and up to December 2020, more than 420 000 people were diagnosed with the disease (Stirioficiale.ro, 2020). On the 16th of March, the president declared a state of emergency, thus imposing various restrictions on the population. The schools were closed, many businesses worked from home or with a reduced schedule, while others suspended the activity altogether. Also, the movement of people was vastly restricted during the day and forbidden during the night. Moreover, religious rites were not permitted during Easter. These measures relaxed after 2 months when the state of emergency was replaced with a state of alert. Although the wearing of masks in closed spaces was mandatory and many businesses still worked from home, the stay-at-home orders were suspended, and the lockdown period ended (Ceauşu, 2020). Globally, from its late 2019 emergence until December 2020, Covid-19 has infected more than 70 million people (World Health Organization, 2020). However, the number of people affected by the perils of the disease is much larger. People live with the fear of getting ill, losing their jobs, and weakening social relationships. Moreover, these challenges do not have an impact on the individual only, but on the family altogether (Panzeri et al., 2020; Spinelli et al., 2020; Overall et al., 2021).

The current crisis has already affected people's mental health, social relationships and family functioning, leading to higher levels of depression, anxiety and stress and decreased social and family activities (Williamson, 2020; Zhang et al., 2020). According to the Vulnerability-Stress-Adaption (VSA) model (Karney and Bradbury, 1995), several factors might influence the decrease of marital satisfaction, and, among them, we can find external stress as well as various preexisting vulnerabilities. Moreover, Pietromonaco and Overall (2020) propose that the current pandemic creates even *more external stressors* that can impact the dyadic relational processes and create further instability inside the couple. The authors mentioned that, in addition to the health-related risks, many faced the risk of losing their jobs, experienced economic strain due to salary reductions, and had to take care of their children on a full-time basis. Moreover, the quarantine, although beneficial for one's health and for preventing the spread of the disease, created even more problems for the couples, such as increased negativity, hostility, and withdrawal (Pietromonaco and Overall, 2020).

Past research showed that significant negative life events, such as wars and medical crises, exacerbate preexisting levels of stress and might lead to higher chances of relational dissolution (Prime et al., 2020). We also know that previous studies linked the stressors associated with the Covid-19 pandemic and the lockdown period, such as social isolation, financial strain or fear of Covid-19, with decreased marital satisfaction (Balzarini et al., 2020; Reizer et al., 2020; Schmid et al., 2021). Building from the first two models and from the existing empirical evidence, we can assume that the levels of stress in the context of the Coronavirus pandemic and lockdown would be associated with lower levels of relational satisfaction. However, these studies only explored the role of one partner's felt stress on their relational satisfaction. The changes associated with the pandemic disrupt the functioning of the whole family and the stress that disturbs one individual can have negative effects on the partner too (Prime et al., 2020). Thus, with this study, we aimed to explore both the actor associations (the way the stress of one partner is related to his/her satisfaction) as well as the partner associations (the way the stress of one partner is related to the other partner's satisfaction). Therefore, by employing the actor-partner interdependence model (Cook and Kenny, 2005), we proposed the following hypothesis:

H1. A higher level of external stress felt at the beginning of the lockdown by one partner would be associated with lower levels of their own and their partners' marital satisfaction after the lockdown ended. We expected this hypothesis to be met for both men and women.

Stressful events are not seen by a husband or a wife only as a personal burden, but as one that also affects their relationship (Randall and Bodenmann, 2017). According to Bodenmann (1995) systemic-transactional model (STM), the stressors that originate outside of the relationships can spillover into the relationship, generating internal stress. Together, these two types of stress are related to important drops in the quality of romantic relationships (Randall and Bodenmann, 2009). The spillover of stress affects relational satisfaction through multiple mechanisms, such as decreasing time spent together by the partners, weakening the feelings of mutuality, decreasing communication, or increasing the chance that some problematic traits (anxiety, depression, rigidity) will appear (Bodenmann, 2000). Various studies from recent years support this theoretical framework by showing that external stress determines an increase in internal, relational stress (Ledermann et al., 2010; Falconier et al., 2014) and a decrease in marital satisfaction (Hilpert et al., 2013; Backes et al., 2016; Bahun and Huić, 2017). Moreover, other studies show that these effects are stable over time (momentary stress affects subsequent marital satisfaction) and that the level of stress perceived by one partner can impact both their satisfaction and their partner's satisfaction (Neff and Karney, 2004; Falconier et al., 2014; Rusu et al., 2020).

To our knowledge, no study has verified this spillover effect during the Covid-19 pandemic. Still, according to Pietromonaco and Overall's (2020) model, external stress can lead to maladaptive dyadic processes such as negativity and hostility. With this study, we aimed to explore whether external stress is associated with marital satisfaction through internal stress. Thus, both theoretical and empirical evidence (Ledermann et al., 2010; Falconier et al., 2014) support the following hypothesis:

H2. Each partner's external stress will have an indirect negative association with their own marital satisfaction and with their partner's marital satisfaction through each partner's levels of internal stress.

Moreover, the pandemic, as well as the lockdown period, might be particularly damaging for the families with lower socioeconomic status (SES), compared to those with a higher socioeconomic status. Contextual vulnerability might increase the effects of stress during the pandemic. According to Pietromonaco and Overall (2020), socioeconomic status, as an indicator of social class, acts as an important vulnerability, exposing the couples to even higher levels of stress. Through the lenses of cultural psychology, social class can take a subjective perspective (the subjective perception of social rank in relationships to others) or an objective one (measured through education or socioeconomic status) (Grossmann and Na, 2013). Moreover, the objective social class “may act as culture *per se*, acquired and shaped in interaction with the class-typical environment, and via the socialization of class-related practices” (Grossmann and Huynh, 2013, p. 113). Indeed, previous studies have shown that lower socioeconomic status (SES), as indicated by lower income, was associated with higher levels of stress (Baum et al., 1999; Yang et al., 2017) and, regardless of the country's GDP, with lower levels of relational satisfaction (Dobrowolska et al., 2020). Stressful contexts, such are those experienced by the couples with lower SES, hinder positive interactions between the partners, and exacerbate the problems with the relations (Neff and Karney, 2017). Some authors in the field of cultural psychology even argue that a lower SES determines different patterns at cognitive, emotional, and behavioral levels (Grossmann and Huynh, 2013). Starting from a model by Kraus et al. (2009) and Manstead (2018) argues that people with lower SES have low perceived control over their environment, make more situational attributions, and have their focus on others and on interdependence, compared with those with higher SES, who have a higher perceived control, make mostly dispositional attributions and focus on themselves and on their independence. Having lower incomes, people with lower SES also have reduced influence on others and on their environment. Thus, they have a limited ability (objective and subjective) to affect future outcomes, which translates into a lack of perceived control (Pepper and Nettle, 2017). Taking this into account, they become increasingly reliant on those around them and on the social contexts, which increases their levels of interdependence. On the contrary, people with higher SES reinforce their independent cultural ideas, try to stand out from others, and to influence their social contexts (Stephens et al., 2014). Such differences can be crucial during the Covid-19 pandemic. Due to social distancing and isolation, people might have trouble contacting their friends and peers (Pietrabissa and Simpson, 2020). By negating their usual reliance on others, these factors might further affect their levels of stress, adaptation, and family functioning for those with lower SES. Moreover, they might feel particularly threatened by this adverse context which, in turn, can accentuate their lack of control over the situation. For the individuals with higher SES, their position during the pandemic, although harsh, might not be as dire. They have lower chances to be affected from a financial standpoint, they are less reliant on others and more self-focused, which can protect them more against stress. Indeed, during the Covid-19 pandemic, factors such as poverty and unemployment were associated with increased Covid-19 diagnosis and mortality (Khazanchi et al., 2020). Moreover, working-class individuals, those with occupations that require more interpersonal contact and that cannot be performed remotely, had more chances of losing their jobs compared to those with better paid jobs, who can work from home (Montenovo et al., 2020). Also, recent research suggested that during the Covid-19 pandemic, people with lower SES, such as those without work and those with lower income, report increased levels of depression and anxiety compared to the pre-pandemic period (Hamadani et al., 2020, Pieh et al., 2020). Based on this previous work, we aimed to test whether there are differences based on SES in the models specified for Hypotheses 1 and 2. We formulated the following hypotheses:

H3. We expected that the negative effects of stress on relationship satisfaction would be stronger for those with lower SES than those with higher SES.

Finally, another potential contextual vulnerability when facing the threats of the Covid-19 pandemic is gender. Previous research showed that women, compared to men, have more chances of losing their job and facing depression during the pandemic (Dang and Nguyen, 2020; Pieh et al., 2020). Also, during the pandemic, women report higher levels of emotional and physical violence compared to men (Patel et al., 2020). Thus, for women, the factors contributing to higher stress during the pandemic could be more numerous. It is also worth noting that women generally score higher than men in chronic and daily stress (Pilar Matud, 2004), and lower in marital satisfaction (Jackson et al., 2014). The actor-partner interdependence model (Cook and Kenny, 2005) allows us to explore the pathways from stress to marital satisfaction separately for men and women.

To assess the psychological effects of Covid-19, longitudinal studies are needed to compare the results at the beginning, during, and at the end of the pandemic. Moreover, it is important to differentiate between the periods of stay-at-home lockdown and those when people are allowed to go outside without or with minimal regulations. Our study uses data gathered during two waves at the beginning of the Covid-19 pandemic in Romania. The first set of data was gathered during the first days of the lockdown (the middle of March 2020) and the second set was gathered after the lockdown was suspended (the middle of May 2020). This data allows us to investigate longitudinal effects on marital satisfaction by taking into account both the pandemic and the lockdown.

## Materials and Methods

### Procedure

The study's procedure was approved by the University Ethical Committee. Participants were then recruited with the help of undergraduate students enrolled at a north-eastern Romanian university. The students were asked to distribute the questionnaires to couples that were married for at least 1 year immediately after the national lockdown was instituted (after the 16th of March, 2020). The questionnaires were distributed using an online form and contained demographic measures and the scales for internal and external stress and couple satisfaction. The participants agreed to fill in the questionnaires voluntarily and were not rewarded for their participation. 204 couples returned their questionnaires. From these, 5 couples were not married and had relationships shorter than 1 year were eliminated from the study because they did not meet the inclusion criteria. After the lockdown ended (15th of May, 2020) the couples were contacted again by email and asked to complete a second set of instruments, containing the measure for couple satisfaction. Only 144 couples returned their questionnaires. The participants declining enrolment in the second wave of the study did not offer a reason. They were, however, relatively equally distributed across SES levels (29 from the higher SES group and 26 from the lower SES group).

### Participants

The sample consisted of 144 heterosexual married couples (*N* = 288 individuals). During the first wave, women had a mean age of 43.32 years (SD = 9.35, range 25–76) and men of 45.10 years (SD = 10.11, range 25–82). On average, the marriage duration was ~18.75 years (SD = 10.31 years; range 1–55 years). The average number of children per household was 1.47 (SD = 0.9; range 0–5). Among women, 10 participants declared they were not working at the time of the survey. Among men, three participants were retired at the time of the survey. All the other participants were employed in the first wave of the survey. In the second wave, all participants reported having the same professional status.

### Measures

#### Internal and External Stress

Each participant's levels of internal stress (coming from inside the relationship) and external stress (coming from outside the relationship) were assessed with the *Multidimensional Stress Questionnaire for Couples* (MSQ-C; Bodenmann, 2006). The internal stress subscale consists of 10 items rated on a 4-point Likert-type scale (ranging from 1 = not at all to 4 = highly stressful) and measures the level of stress caused by the situation originating within the couple's relationship over the last 7 days. The items demonstrated a good internal consistency (α men T1 = 0.91; α female T1 = 0.91). External stress from daily hassles was measured using an 8-item subscale. Respondents rate how stressful daily situations outside their couple have been over the past 7 days on a similar 4-point Likert-type scale. The internal consistency of this subscale was also good (α men T1 = 0.86; α female T1 = 0.79). For both scales, the participants were asked to take into account the context they were in (the Covid-19 pandemic and lockdown). For each scale, the total score is computed by averaging the responses offered to each item. Higher total scores indicate higher levels of external, respectively internal stress.

#### Marital Satisfaction

The partners' satisfaction level was measured using the *Couple Satisfaction Index 4* (CSI 4, Funk and Rogge, 2007). This is the short version of a 32-items instrument that assesses an individual's level of satisfaction with their romantic relationship. The CSI was created by selecting the best items from the already existing measures of satisfaction. Respondents indicated how content they feel in their marital relationship on a 7-point Likert scale for one item and a 6-point Likert scale for the others. The items demonstrated a good internal consistency (α men T1 = 0.80, α men T2 = 0.88; α female T1 = 0.86, α female T2 = 0.87). The total score is computed by summing up the responses offered to each item. On the resulting continuous scale, higher total scores indicate higher levels of marital satisfaction.

#### Socioeconomic Status

SES was measured through the monthly household income, which was assessed by the following question: “The total household income of your family is: (a) <2,500 lei; (b) between 2,500 and 5,000 lei; (c) between 5,000 and 7,500 lei; (d) more than 7,500 lei.” These categories were created based on the average monthly household income at the time of the survey, which was about 5 100 Romanian lei (~1,000 euros; Institutul National de Statistică, 2020). For all the couples, the partners offered similar answers. Based on their answers, two levels of household income were created: 5,000 lei ore less (1), and more than 5,000 lei (2). 63 couples (43.8%) reported a monthly household income lower than 5,000 lei and 81 couples (56.4%) reported a monthly household income higher than 5,000 lei. Thus, the former couples were considered as having lower SES and later couples as having higher SES.

#### Demographic Data

Each partner completed information about gender, age, the length of the marriage, marital status, number of children, professional status, and current household income.

### Data Analysis

The preliminary analyses and the Pearson correlations between the variables were conducted using the SPSS 21 software. To verify the hypotheses, we tested a multigroup mediation model using SEM with the IBM SPSS AMOS, version 21.0. The partners' external stress levels measured at T1 were entered as predictors and their marital satisfaction measured at T2 as outcomes. We also used the partners' internal stress levels at T1 as mediators. The model fit was assessed based on chi-square, comparative fit index (CFI > 0.90), Goodness of Fit Index (GFI > 0.90), root mean square error of approximation (RMSEA < 0.06–0.08), and the standardized root mean square (SRMR < 0.08). We used a sample of 5,000 for bootstrapping and a 95% confidence interval (CI), where the absence of zero indicates a significant effect. In the end, for marital satisfaction, we compared the total variance accounted for by the four stress variables (one's own external and internal stress and the partner's external and internal stress) in each group (low SES vs. high SES). This analysis, computed separately for men and women, was conducted using a *z*-score test where we compared the multiple regression coefficients.

## Results

### Preliminary Analyses and Correlation Analyses

The means, standard deviations and correlations are presented in Table 1. We analyzed the zero-order correlation between men's and women's internal stress, external stress, and marital satisfaction separately for those in the low socioeconomic status group as well as for those in the high socioeconomic status group (see Table 1). For both groups, we found that men's and women's levels of satisfaction were strongly and positively associated. Also, women's external stress was associated with their partner's and with their own and their partner's internal stress and marital satisfaction (at T2). In the high SES group, men's external stress was associated with their own and their partners' internal stress. For the low SES group, the same results were found, but in addition, the men's external stress was associated with their own and their partners' marital satisfaction at T2. Men's and women's internal stress was associated with their partner's and with their own and their partners' marital satisfaction at T2 in both groups.

**Table 1:** Means, Standard deviations and correlations among the variables for low SES participants (*N* = 63, below the diagonal) and high SES participants (*N* = 81; above the diagonal).

	**M**	**SD**	**1**	**2**	**3**	**4**	**5**	**6**	**7**	**8**	**M**	**SD**
1. E Stress W T1	1.90	0.71		0.60^***^	0.50^***^	0.47^**^	−0.31^**^	−0.38^***^	−0.32^**^	−0.29^**^	1.77	0.48
2. E Stress M T1	1.90	0.70	0.61^***^		0.38^***^	0.69^***^	−0.21	−0.33^**^	−0.05	−0.15	1.63	0.58
3. I Stress W T1	1.96	0.65	0.56^***^	0.44^***^		0.59^***^	−0.61^***^	−0.58^***^	−0.49^***^	−0.46^***^	1.71	0.63
4. I Stress M T1	1.68	0.59	0.40^**^	0.56^***^	0.68^***^		−0.34^**^	−0.51^***^	−0.24^*^	−0.30^**^	1.62	0.59
5. Sat. W T1	15.95	3.84	−0.28^*^	−0.18	−0.65^***^	−0.44^***^		0.57^***^	0.75^***^	0.60^***^	16.87	2.95
6. Sat M T1	16.93	3.37	−0.37^**^	−0.34^**^	−0.57^***^	−0.59^***^	0.72^***^		0.44^***^	0.66^***^	17.86	2.70
7. Sat W T2	15.93	3.37	−0.41^**^	−0.26^*^	−0.71^***^	−0.60^***^	0.72^***^	0.71^***^		0.50^***^	17.04	2.57
8. Sat M T2	16.55	3.90	−0.41^**^	−0.37^**^	−0.55^***^	−0.53^***^	0.53^***^	0.71^***^	0.69^***^		17.74	2.74

*** p < 0.001,

** p < 0.01,

* p < 0.05;

*E Stress, External stress; I Stress, Internal stress; Sat, marital satisfaction; W, women; M, Men*.

We also analyzed group differences in the level of each variable. A series of Independent sample *T*-Tests showed that women with lower SES reported a higher level of internal stress at T1 compared to those with higher SES (*t*_(142)_ = 2.25, *p* = 0.02, *d* = 0.39), while male with lower SES reported higher levels of external stress at T1 compared to those with higher SES (*t*_(142)_ = 2.47, *p* = 0.01, *d* = 0.40). Although at T1 we did not find any significant differences regarding marital satisfaction, during the second assessment, both men (*t*_(142)_ = −2.15, *p* = 0.03, *d* = 0.35) and women (*t*_(142)_ = −2.24, *p* = 0.02, *d* = 0.37) with lower SES reported lower satisfaction compared to those with higher SES.

We were finally interested in exploring gender differences. After conducting a serios of Paired Sample *T*-Tests, we observed that, regardless of their socio-economic status, women reported more internal stress than men at T1 (*t*_(143)_ = 3.87, *p* < 0.001, *d* = 0.27), and that men reported higher levels of satisfaction comparted to women during the first (*t*_(143)_ = −4.77, *p* < 0.001, *d* = 0.30) and second assessment (*t*_(143)_ = −2.91, *p* < 0.01, *d* = 0.20). We found no gender differences regarding external stress at T1 (*t*_(143)_ = 1.87, *p* = 0.06, *d* = 0.14).

### Hypotheses Testing

Next, we conducted a multi-group structural equation model analysis with mediation to test whether internal stress can explain the relationship between external stress and marital satisfaction and to explore the moderating role of socioeconomic status. This allowed us to simultaneously test the relationships between external stress, internal stress, and marital satisfaction, as well as the mediating role of internal stress for both groups. In this model, we allowed the control variables (men's and women's marital satisfaction at T1) to correlate with all the other variables. Also, men's and women's similar variables were allowed to correlate between them. The unconstrained model presented the following indices: χ2 = 4.59, df = 2, *p* = 0.101, GFI = 0.99, CFI = 0.99, SRMR = 0.002, RMSEA = 0.09. RMSEA was higher the threshold of 0.08, but model with low degrees of freedom tend to offer artificially large values for the measure (Kenny et al., 2015). Taking all these into account, we consider that the indices indicate a good fit. Next, we tested whether restraining all the regression paths across the groups would lead to a significant decrease in model fit. The chi-square difference test showed that the fully constrained model had a worse model fit (*p* = 0.02). As such, we assumed that there were differences between the two groups.

**Figure 1:**
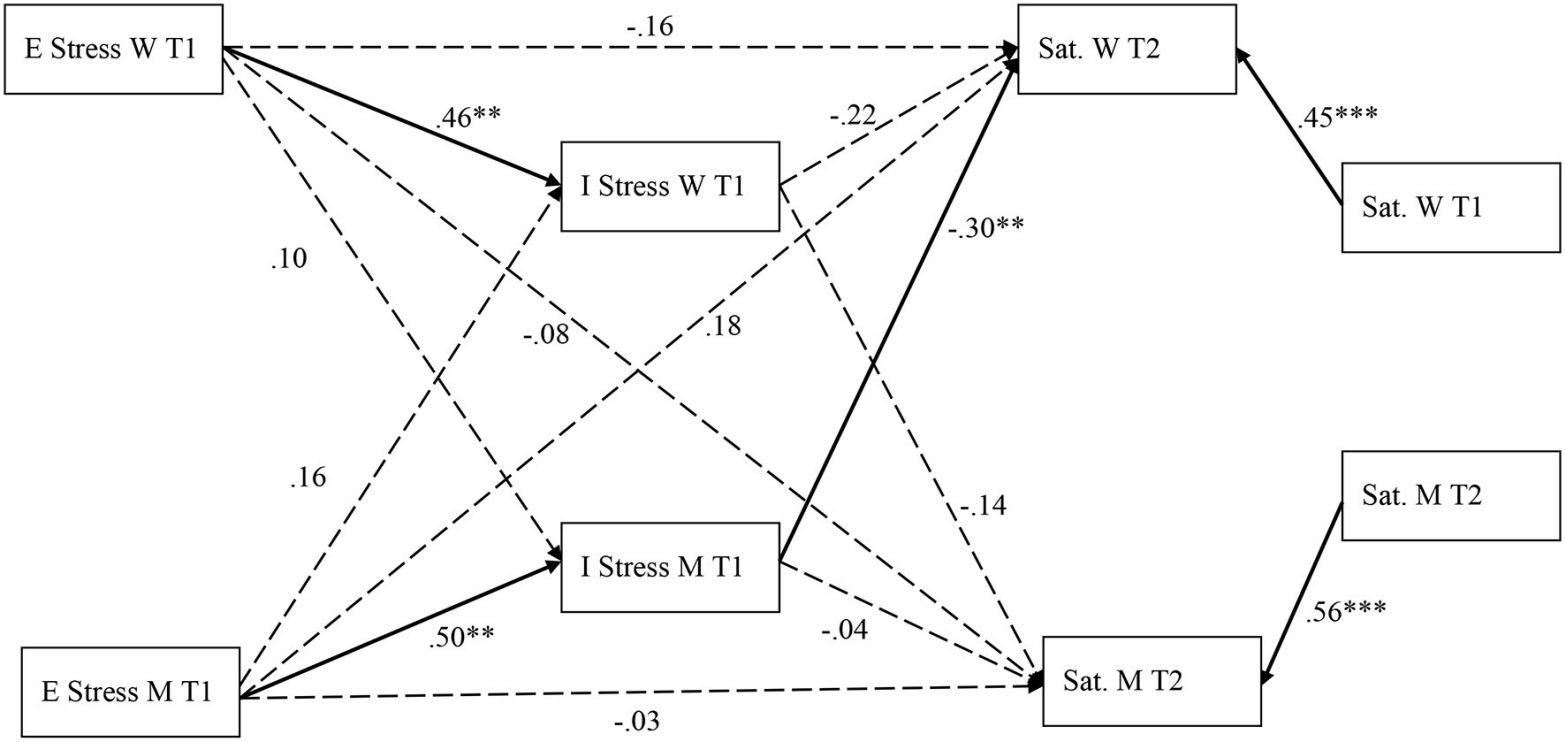
Standardized path estimates for the *low SES group*'s mediation model. ***p < 0.001, **p < 0.01; E Stress, External stress; I Stress, Internal stress; Sat, marital satisfaction; W, women; M, Men. Bolded arrows represent significant paths. Dashed arrows represent non-significant paths.

**Figure 2:**
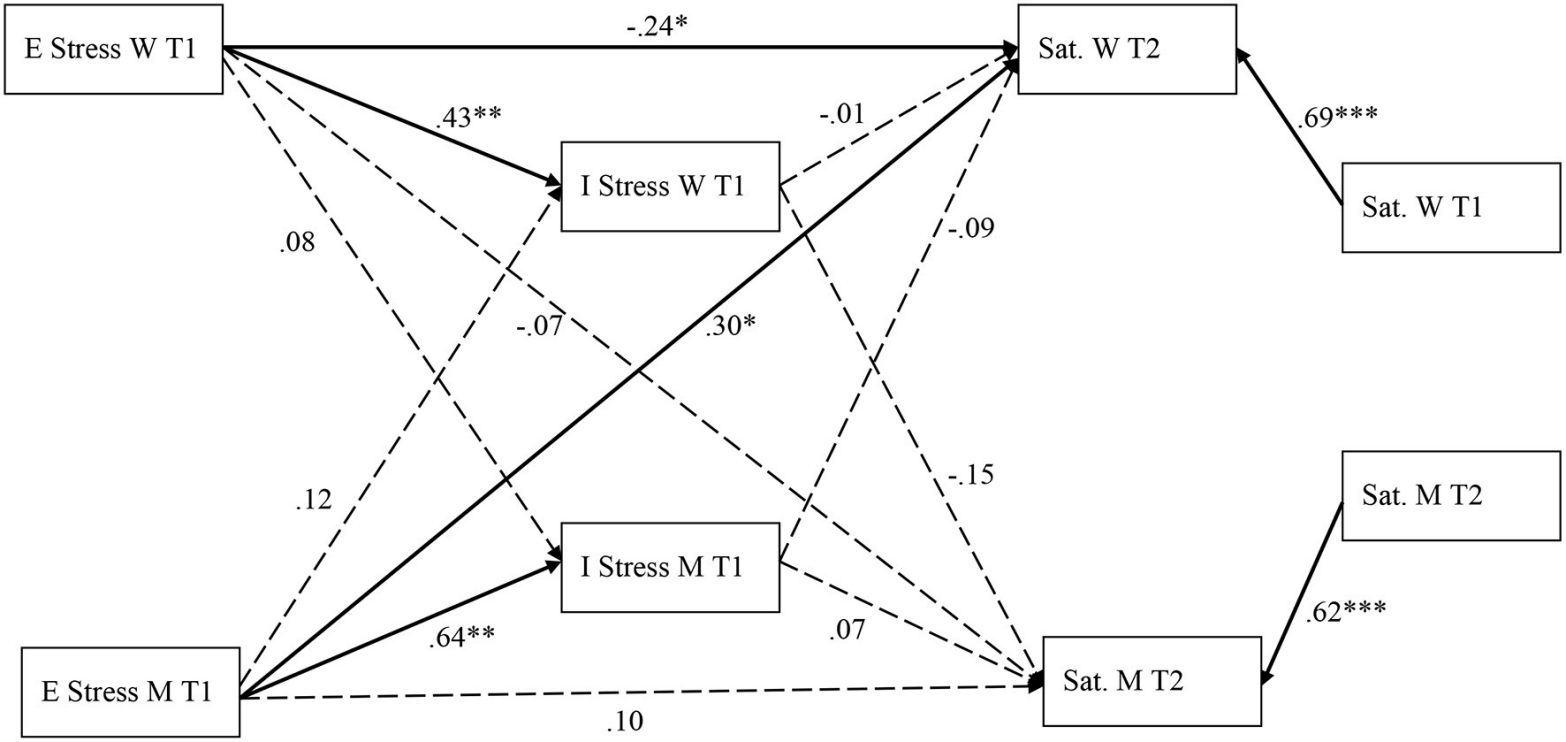
Standardized path estimates for the *high SES group*'s mediation model. ***p < 0.001, **p < 0.01, *p < 0.05; E Stress, External stress; I Stress, Internal stress; Sat, marital satisfaction; W, women; M, Men. Bolded arrows represent significant paths. Dashed arrows represent non-significant paths.

The results for each group appear in Figures 1, 2. For the low SES group (Figure 1), women's external stress at T1 was not associated with the subsequent marital satisfaction (β = −0.16; *p* = 0.12; 95% CI [−0.35; 0.01]) and men's external stress was not associated with their subsequent level of marital satisfaction (β = −0.03; *p* = 0.80; 95% CI [−0.23; 0.17]). Men's external stress at T1 was not associated with their partner's marital satisfaction at T2 (β = 0.18; *p* = 0.10; 95% CI [−0.006; 0.35]). Women's external stress was not associated with their partners' marital satisfaction at T2 (β = −0.08; *p* = 0.50; 95% CI [−0.30; 0.12]).

Women's (β = 0.46; *p* = 0.001; 95% CI [0.24; 0.66]) and men's (β = 0.50; *p* = 0.001; 95% CI [28; 0.69]) levels of external stress at T1 were associated with their own levels of internal stress, but not associated with their partner's levels of internal stress (for women: β = 0.10; *p* = 0.47; 95% CI [−0.14; 0.31]; for men: β = 0.16; *p* = 0.22; 95% CI [−0.07; 0.37]). Internal stress was not associated with their own marital satisfaction at T2 for either women (β = −0.22; *p* = 0.13; 95% CI [−0.42; 0.28]) or men (β = −0.04; *p* = 0.80; 95% CI [−0.27; 0.19]). Finally, only men's internal stress at T1 was associated with women's marital satisfaction at T2 (β = −0.30; *p* = 0.01; 95% CI [−0.48; −0.15]), while women's internal stress was not associated with men's marital satisfaction (β = −0.14; *p* = 0.28; 95% CI [−0.36; 0.08]). In regards to the proposed mediation, we found one significant indirect effect. Men's internal stress at T1 mediated the relationship between their external stress at T1 and their partners' satisfaction at T2 (β = −0.18; *p* = 0.01; 95% CI [−0.32; −0.09]). Given that the direct effect of men's external stress of women's marital satisfaction was not significant, we can consider that men's internal stress fully mediated this relationship. For the low SES group, one's own external and internal stress, as well as the partner's internal and external stress explain 67% of the variability of women's marital satisfaction and 55% of the variability in men's marital satisfaction.

For the high SES group (Figure 2) and in contrast to the low SES group, women's external stress at T1 was associated with their marital satisfaction at T2 (β = −0.24; *p* = 0.02; 95% CI [−0.39; −0.06]), which was not the case for men (β = 0.10; *p* = 0.41; 95% CI [−0.11; 0.32]). In regards to the partner effects, only men's external stress was associated with their partners' marital satisfaction at T2 (β = 0.30; *p* = 0.01; 95% CI [0.10; 0.49]. Women's external stress was not linked to men's marital satisfaction at T2 (β = −0.07; *p* = 0.01; 95% CI [−0.32; 0.10]).

For both women and men, external stress was associated with their own internal stress (for women: β = 0.43; *p* = 0.002; 95% CI [0.21; 0.62]; for men: β = 0.64; *p* = 0.001; 95% CI [0.49; 0.78]). Women's external stress was not associated with their partners' internal stress (β = 0.08; *p* = 0.42; 95% CI [−0.08; 0.45]). A similar result was found for men (β = 0.12; *p* = 0.28; 95% CI [−0.07; 0.28]). Internal stress was not associated with marital satisfaction at T2 for either women or men (for women: β = −0.01; *p* = 0.90; 95% CI [−0.20; 0.17]; for men: β = 0.07; *p* = 0.58; 95% CI [−0.14; 0.30]. Women's internal stress was not associated with men's marital satisfaction (β = −0.15; *p* = 0.22; 95% CI [−0.35; 0.05]). In contrast to the low SES group, the relationship between men's internal stress at T1 and women's marital satisfaction at T2 was no longer significant (β = −0.09; *p* = 0.40; 95% CI [−0.28; 0.09]). In addition, we found no significant indirect effect of men's external stress at T1 on women's marital satisfaction at T2, through men's internal stress at T1 (β = −0.06; *p* = 0.37; 95% CI [−0.19; 0.05]). For the higher SES group, one's own external and internal stress, as well as the partner's internal and external stress account for 61% of the variability of women's marital satisfaction and 46% of the variability in men's marital satisfaction.

Finally, for marital satisfaction, we compared the total variance accounted for by the four stress variables. For women, as well as for men, we found no significant difference between the low SES group and the high SES group (women: *z* = 0.47, *p* = 0.31; men: *z* = 0.81, *p* = 0.20).

## Discussion

The Covid-19 pandemic brought up an increase in the number of stressors that can affect the functioning of a family. Thus, the first aim of this study was to examine the associations between external stress during the pandemic and the levels of marital satisfaction for the partners. Moreover, external stress can spillover and affect the internal processes within the family. The second aim was to verify whether internal stress mediated the previous associations. Finally, some individuals and couples are more vulnerable than others when facing stress. By applying the framework of cultural psychology, we aimed to examine how socioeconomic status (SES), here measured through the level of household income, moderated the links between stress and marital satisfaction. To increase the usefulness of our results, we used longitudinal data and conducted the analyses at a dyadic level.

We found that, among the individuals with higher SES, women's external stress at the beginning of the pandemic was negatively associated with their own level of marital satisfaction after 2 months (after the lockdown period was suspended). However, the same relationships were not significant for women with lower SES and for men, regardless of their SES. These findings offer only partial support for our first hypothesis. These gender differences are in contradiction to the results found on couples facing stress in more ordinary circumstances (Randall and Bodenmann, 2017). Still, other studies have shown that women are more susceptible than men to the damaging psychological impact of the pandemic. Women around the world were more vulnerable to stress, anxiety, and depression (Limcaoco et al., 2020), and also to higher levels of worry, and fear of Covid-19 (Bakioglu et al., 2020). Moreover, women have higher risks of losing their jobs during the pandemic (Dang and Nguyen, 2020). Interestingly, men reported higher satisfaction at both time points. Similar differences were found by Rusu (2016) and by Marginean et al. (2010, as cited in Rusu et al., 2018) who reported that women suffer from lower marital and life satisfaction as opposed to men.

In both groups, the partners' external stress seems to spillover and increases their internal stress, which confirms that the STM model (Bodenmann, 1995) is relevant during the Covid-19 pandemic. However, internal stress did not predict satisfaction for either men or women, thus the proposed actor effects were not significant. A possible explanation for this is that the lockdown period provided couples with more time together, which could have improved their levels of closeness and collaboration, which might have led to a non-significant association with marital satisfaction. Future studies are needed to test this supposition.

For couples with higher SES, men's higher external stress was associated with their partners' higher marital satisfaction. This suggests that, among the individuals with higher SES, women's marital satisfaction is linked not only to their own levels of stress but also to their partners'. However, this effect is surprising and challenging to explain. One possible mechanism that explained the results might be offered by the use of coping. Some studies show that higher levels of stress can be linked to increased performance, especially when it is challenge-oriented, and successfully coping with stress can lead to personal growth and self-confidence (Lepine et al., 2005; Liu et al., 2017). Using these findings, we may assume that women, when faced with their husbands' external stress, may feel more helpful and deployed more efficient coping strategies that, over time, lead to some increases in their level of marital satisfaction. These results could also be explained by the differences in independence and perceived control that differentiates individuals with higher and lower SES. Firstly, women with higher SES might be more autonomous. Secondly, they feel they have more control over their environment. Previous research pointed out that people with higher levels of autonomy and lower levels of control orientations also use less defensive coping and self-handicapping strategies (Knee and Zuckerman, 1998). Thus, higher SES women might use less negative coping and more positive coping, which negates the negative effects of the husband's stress on their satisfaction. These proposed mechanisms should, however, be tested by future studies.

We also found evidence for the mediating role of men's internal stress in the relationship between men's external stress and women's marital satisfaction. This link was significant only for families with lower SES. This full mediation is easier to explain, as it supports STM and Pietromonaco and Overall's (2020) model showing that external stress can lead to increased maladaptive dyadic processes. This finding also supports the crossover model of stress proposed by Westman (2001). This process occurs when the stress experienced by one partner affects the satisfaction of the other partner. The crossover process can transpire through empathy or through some mediating mechanisms. By considering this model and the gender difference between men and women, we can propose a possible explanation for our findings. Firstly, women are more emphatic (Eisenberg and Lennon, 1983). Thus, supposedly, they can be more reactive to their partner's internal stress. Secondly, men are more prone to criticize, blame the partner, and provide inconsiderate advice on the days when they are stressed (Neff and Karney, 2005) which can act as a possible mediation mechanism between men's stress and women's marital satisfaction. Finally, the difference in SES might also account for these findings, which contrast with the ones found for higher SES couples. Given their limited access to resources, higher material constraints and lower levels of control, people from low SES backgrounds are more likely to be socialized to view themselves as embedded in close relationships and thus, as more interdependent with close others (Stephens et al., 2014). This allows us to speculate that women from low SES couples, compared to those from high SES couples, could be more aware of the partners' internal struggles and also more affected by them.

We found that stress explains similar proportions of variability in the participants' level of marital satisfaction, regardless of their SES levels, which made us reject the third hypothesis. However, despite not using formal comparison tests, our analyses suggest the possibility that the links between external stress, internal stress and marital satisfaction are different based on the SES level. Moreover, we found that men with lower SES experience greater external stress, women with lower SES experience more internal stress, and all the participants with lower SES had lower levels of marital satisfaction at the second assessment. These results can be explained, at least partially, by the fact that higher SES individuals have superior financial stability and are less affected by the risk created by the pandemic. Although their lifestyle also changed, they still had lower chances of losing their jobs and suffering important financial blows. On the contrary, many factories and small businesses (restaurants, small shops) closed their gates during the pandemic; this might have put more strain on the working-class individuals. The pandemic brought more uncertainty for the people that were already vulnerable in the face of economic hardships which resulted in higher levels of stress and lower satisfaction. However, social classes (as they are defined by the level of SES) also present differences in their social orientation and cognitive styles, as well as in the development of self. Individuals from a lower social class tend to put greater emphasis on interdependence, being more focused on relatedness and social connection (Grossmann and Na, 2013). But during the early days of the pandemic and of the lockdown period, the authorities insisted on many social restrictions, such as distancing, quarantine, and isolation. For the people that benefited more from social connections, reducing said social connection might be increasingly threatening and might affect mental health (Leigh-Hunt et al., 2017). On the contrary, higher social class individuals see themselves are more independent, more separate from social others, and might be less impacted by isolation and loneliness. Moreover, lower-class individuals tend to explain social outcomes by using more contextual factors (that are outside of their control), as contrasted to higher-class individuals who use more dispositional (and controllable factors) (Kraus et al., 2009). Thus, feeling that their environment is less controllable during the pandemic, lower social class individuals might develop an increased sense of helplessness (Soral et al., 2021). In the end, some past reviews suggest that socioeconomic status and gender are two sociocultural contexts the determine the elaboration of one self over the others (Stephens et al., 2014). Some of our results suggest that socioeconomic status and gender, being responsible for the development of a self that acts interdependently in more numerous situations, might lead to higher vulnerability during the Covid-19 pandemic. However, this possibility should be tested in future studies.

### Practical Contributions

This study uses longitudinal data and shows that higher levels of stress on the part of both partners are negatively linked to marital satisfaction during the lockdown. Moreover, some of our results suggest that not all families respond the same when facing stress in the context of the Covid-19 pandemic and lockdown and that some are more protected than others. These findings provide valuable information for therapists, health professionals, policy makers, and researchers. Given that these outcomes might be determined by their preexisting vulnerabilities, as well as by the importance they put on social relationships, the solution must be found not only on the economic level but also on a social one. Improving social support was already highlighted by Saltzman et al. (2020) as a possible mechanism with great health benefits during the Covid-19 pandemic. But it seems that some people are more dependent on social support compared to others and policymakers could pay increased levels of attention to them. Moreover, the perceived lack of control might also be detrimental to the personal and marital well-being of lower SES couples. Although mitigating negative behaviors and cognitions that are acquired after years of socialization might be difficult, better information and more institutional help could make lower SES individuals feel safer and less exposed to the pandemic.

Not only that this study provides information regarding the families with lower SES, which is considered a risk group that received less attention during this situation (Holmes et al., 2020), but it also suggests that women's satisfaction is more strongly linked to stress, although they also can find some benefits in this situation. We know that women are uniquely influenced by the pandemic and more exposed to stress and negative mental health (Reizer et al., 2020) but this study suggests that they are not necessarily more prone to dips in satisfaction. However, their satisfaction is impacted by both their levels of stress and by their partner's level of stress. For women with lower SES, their partners' levels of external and internal stress are detrimental to their marital satisfaction. However, for women with higher SES, their own external stress is detrimental to their satisfaction, while their partners' external stress is positively associated with their satisfaction. Although we did not measure coping, this relationship might be mediated by various coping mechanisms that can wear off with time. With this longitudinal study we showed that some women feel more satisfaction at the end of the lockdown when their partners felt more external stress at the beginning, but over longer periods of time, especially in case of another lockdown, this effect might not remain significant. Longer longitudinal studies are needed to explore the role of stress on satisfaction throughout the whole pandemic, for men and women alike.

The Covid-19 pandemic and the restrictions were already associated with negative mental and psychological reactions around the world (Trzebiński et al., 2020), and this study offered supplementary information on how stress is associated with marital satisfaction. We showed that external stressors are linked to higher internal stress and to lower satisfaction. Moreover, we suggest that socioeconomic status might act as a potential vulnerability. In general, our findings support the model proposed by Pietromonaco and Overall (2020). In order to keep the families safe, some measures could be taken. Stanley and Markman (2020) suggested that extensive attention should be given to physical, emotional, commitment, and community safety. Given that women and families with lower SES are uniquely affected by stress, we emphasize the need to pay attention to these foundations of safety.

### Strengths, Limitation, and Future Directions

This study presents a series of noteworthy strengths. Firstly, we used dyadic and longitudinal data to assess family functioning during the Covid-19 pandemic. Dyadic data offer a better view of the functioning of the couple by taking into account how one partner influences the other. Moreover, we used an appropriate and complex analytic strategy by including mediation in our model. Thus, we identified some explanatory mechanisms of the relationships between the partner's levels of external stress during the pandemic and their subsequent levels of marital satisfaction. Longitudinal data offers the possibility to infer some causal relationships between the variables. We also used a multi-group SEM analysis to explore the model based on the socioeconomic status of the participants. This allowed us to investigate whether the characteristics of one culture act as vulnerability for the individuals facing the dangers of Covid-19.

Still, the study is not without its limitations. Firstly, we did not measure the variables directly concerning the pandemic, choosing to concentrate on stress and marital satisfaction in the context of the pandemic. Also, other pandemic/lockdown related covariates (i.e., contacting Covid-19, or following social distancing), as well as other sources of external stress, could have been considered. Secondly, we used a short longitudinal design, with only two measures, relatively near the beginning of the pandemic (at least in Romania). To generalize the data, longer-term results should also be taken into account. Future studies should test the impact of stress even after the lockdown ended. Moreover, after a period with a relative decrease in the number of cases after the lockdown ended in most countries, the impact of the Covid-19 increases again in the latter part of 2020. Thus, it would be interesting to see how couples deal with the prolonged stress caused by the pandemic. Thirdly, although we used SES as an objective measure of social class, we categorized the participants into two distinct groups, which could have affected the variability of our data. Future studies could use SES as a continuous variable, thus offering better distinctions between those with lower and higher levels. Also, a subjective view on social class could be integrated and measured. Finally, seeing social class as a culture has its benefits, but in order to truly observe cultural variation in response to Covid-19, future studies should test these association in multiple countries and on various social classes within those countries.

## Conclusions

This study explored how families respond to stress during the Covid-19 pandemic and whether socioeconomic status moderated the responses. Both romantic relationships' functioning and at-risk groups should receive increased attention in this situation (Holmes et al., 2020; Prime et al., 2020) and with this research, we tried to capture the particularities of both. We found that stress is linked to lower satisfaction. However, the partners' external and internal stress have similar contributions to marital satisfaction regardless of the couples' socioeconomic status. Our results suggest a potential difference between the low SES group and the high SES group in the paths linking stress and marital satisfaction, but future studies are needed to clarify this issue. Moreover, the individuals from the lower SES group suffer from more stress and are less satisfied with their marital relationships compared to those from the high SES group. We suggest that, while it is hard to make a clear distinction based on socioeconomic status, this cultural variable might act as a vulnerability during the crisis. Our results contribute both theoretically and practically to a better understanding of the psychological and relational consequences of the Covid-19 pandemic.

## Data Availability Statement

The raw data supporting the conclusions of this article will be made available by the authors, without undue reservation.

## Ethics Statement

The studies involving human participants were reviewed and approved by Ethical Committee of the Alexandru Ioan Cuza University. The patients/participants provided their written informed consent to participate in this study.

## Author Contributions

MT and OC conceived and designed the study, collected the data, and wrote the manuscript. OC analyzed the data. All authors contributed to the article and approved the submitted version.

## Conflict of Interest

The authors declare that the research was conducted in the absence of any commercial or financial relationships that could be construed as a potential conflict of interest.
